# Improved memory for information learnt before alcohol use in social drinkers tested in a naturalistic setting

**DOI:** 10.1038/s41598-017-06305-w

**Published:** 2017-07-24

**Authors:** Molly Carlyle, Nicolas Dumay, Karen Roberts, Amy McAndrew, Tobias Stevens, Will Lawn, Celia J. A. Morgan

**Affiliations:** 1 Psychopharmacology and Addiction Research Centre (PARC), University of Exeter, Exeter, UK; 2 BCBL, Basque Center on Cognition, Brain and Language, Donostia-San Sebastián, Spain; 3 Clinical Psychopharmacology Unit, University College London, London, UK

## Abstract

Alcohol is known to facilitate memory if given after learning information in the laboratory; we aimed to investigate whether this effect can be found when alcohol is consumed in a naturalistic setting. Eighty-eight social drinkers were randomly allocated to either an alcohol self-dosing or a sober condition. The study assessed both retrograde facilitation and alcohol induced memory impairment using two independent tasks. In the retrograde task, participants learnt information in their own homes, and then consumed alcohol ad libitum. Participants then undertook an anterograde memory task of alcohol impairment when intoxicated. Both memory tasks were completed again the following day. Mean amount of alcohol consumed was 82.59 grams over the evening. For the retrograde task, as predicted, both conditions exhibited similar performance on the memory task immediately following learning (before intoxication) yet performance was better when tested the morning after encoding in the alcohol condition only. The anterograde task did not reveal significant differences in memory performance post-drinking. Units of alcohol drunk were positively correlated with the amount of retrograde facilitation the following morning. These findings demonstrate the retrograde facilitation effect in a naturalistic setting, and found it to be related to the self-administered grams of alcohol.

## Introduction

The marked reduction in the ability to form new memories following drinking, or anterograde memory impairment, is well documented^1^. Paradoxically, however, along with impairing memory for information learnt when intoxicated, alcohol has been found to enhance memory for information learned *prior* to drinking^2^. This alcohol-related memory enhancement means that those who have consumed alcohol exhibit less forgetting of information learned prior to drinking, compared to those who have not consumed alcohol. Evidence supporting this effect, termed ‘retrograde memory facilitation’, has accumulated using a variety of different declarative memory tasks.

Verbal memory paradigms have predominantly been used to investigate this effect. Individuals given a list of words to learn prior to drinking 0.66 ml/kg alcohol showed better free recall performance compared to a placebo group when tested two hours later^3^. In another study, participants were able to recall a larger proportion of a prose narrative that was learned before drinking (1.00 ml/kg) when tested the subsequent morning, compared to those who had not drunk alcohol^4^. The retrograde facilitation is not restricted to verbal memory processes alone, however, as improvements in scene recognition have also been observed when learning was followed by both 0.5 and 1.0 ml of alcohol/kg, but not 0.025 ml, when tested the subsequent morning^2^. Furthermore, research has shown that memory for both positively and negatively valenced statements was enhanced when followed by a drinking session of 1.0 ml of alc/kg, when tested the following day^5^. Although dose varied to a small degree among such studies, they all concluded that participant’s memory for previously learned information is stronger following a period of alcohol intoxication. Importantly, this retrograde facilitation is an enhancement in memory, not just reduced forgetting across the same time frame.

The leading explanation for retrograde facilitation by alcohol is that by blocking learning of new information with alcohol, one will have more resources available to lay down other recently learned information into long term memory, due to an enhancement of memory consolidation. This evidence suggests that when the hippocampus is not encoding new information, it switches to consolidation of recently learnt information^6^. Alcohol can acutely disrupt the processes that underlie memory consolidation by interfering with long-term potentiation (LTP); a central mechanism required for forming new memories^7, 8^. It is thought that alcohol can facilitate consolidation by disrupting the *induction* of LTP for new information, rather than the maintenance of previously induced LTP^9^. This model suggests that alcohol facilitates consolidation by creating periods of reduced encoding, and is the general consensus for explaining the mechanisms behind retrograde facilitation^2, 10^. Thus, anterograde impairments may be important for eliciting retrograde facilitation by causing a reduction in this ‘retroactive interference’^3^. This neurobiological explanation is consistent with previous studies, as they reveal that alcohol causes dose-dependent improvements in memory^2^, and a primacy effect where enhanced recall is more pronounced for the former stimuli learned^3^. Yet despite evidence reporting retrograde facilitation, and the speculation behind its neurobiological underpinnings, this phenomenon has not yet been examined in naturalistic settings.

Since alcohol is the most popular recreational drug worldwide, with an estimated 38.3% of the global population currently using it^11^, investigating the naturalistic effects of alcohol is important in assessing the harms and potential benefits of this ubiquitous substance. Naturalistic designs also enable researchers to investigate a wider range of doses which are reflective of real-life drinking; opposed to the highly regimented laboratory environments where limits on dose are imposed^12^. Being able to investigate the relationship between naturally varying alcohol dose and subsequent retrograde facilitation would lend further support to the notion that this phenomenon is related to alcohol dosing per se, and not a possible non-specific motivational effect. The current study aimed to investigate whether the retrograde facilitation effect can be observed in a naturalistic setting. This was examined by assessing social drinkers’ performance on memory tasks completed before, immediately after, and the morning after a period of naturalistic, self-dosed binge drinking. Two separate tasks were used to investigate both the acute memory impairments caused by drinking, as well as memory consolidation over time. Post-alcohol acute memory impairments were assessed using a simple mnemonic similarity task (hereafter referred to as ‘anterograde task’), which required participants to discriminate between pictures of repeated, similar or novel objects. This task was chosen due to its sensitivity in observing changes in subtle memory deficits^13, 14^. Retrograde facilitation was assessed using a novel word task (hereafter referred to as ‘retrograde task’) requiring participants to learn a set of novel (made-up) words, and memory of these items was tested explicitly using cued recall, which has been shown to be sensitive to sleep-dependent effects on memory consolidation^16^.

We hypothesised that both alcohol and sober groups would show equal performance on the retrograde task immediately before drinking, but the alcohol group would display better performance when tested the morning after drinking relative to the sober participants, as expected by retrograde facilitation. In addition, we aimed to investigate whether the quantities of alcohol consumed are directly related to consolidation by dose and the anterograde task. We hypothesised that the degree of acute alcohol induced memory impairment would be related to retrograde facilitation the next day, in line with theories that it is the reduction in encoding when intoxicated that produces this fascinating effect.

## Method

### Design and participants

Eighty-eight social drinkers (31 males; 57 females) aged between 18 and 53 years (*M* = 23.26, *SD* = 8.31) were recruited via word of mouth and snowball sampling in a mixed between and within subjects design. Participants were randomly allocated to either the alcohol or sober condition, and both conditions completed all subsequent assessments. Both conditions were matched for gender, and all assessments were counterbalanced across participants. Testing took place in a quiet room in participants’ homes. Although there are other environments that may be more typical of drinking behaviour (such as bars and clubs), this environment was chosen due feasibility constraints.

Participants were required to be: social drinkers, native UK-English speakers, and were absent of any language or auditory impairments. Exclusion criteria were: diagnosis of a severe alcohol use disorder using DSM 5 criteria; a relevant physical or psychiatric illness; a neurological condition; taking regular prescribed medications excluding the oral contraceptive; pregnancy or possibility of pregnancy; a body mass index (BMI) smaller than 16 or larger than 35. All participants provided written, witnessed and informed consent. The study was approved by the University of Exeter Ethics Committee. All methods were performed in accordance with the relevant guidelines and regulations, including the Declaration of Helsinki (2013).

### Measures

#### The retrograde task

To investigate retrograde facilitation, a novel word learning task^16^ was used, which involved two phases that were implemented using the programme DMDX Version 5.1.0.0 with stimuli presented using Sennheiser HD201 over-ear headphones. Participants first completed the learning phase, which involved listening to 24 novel words repeated 36 times in different orders. Participants monitored whether the words contained a target sound (such as ‘n’ in ‘gun’) by pressing a key as quickly as possible. The novel words were similar to existing words, but they contained extra letters, for example ‘frenzylk’. Participants were informed that they would be tested on these novel words later, which occurred immediately after learning and the following morning (session two).

During the second phase, participants completed the cued recall test. This was an explicit measure of how well the new words were remembered. Participants listened to the beginning of the learned 24 novel words (the cue), and were required to give typed responses for the sound missing at the end using two keys. For example, the cue for ‘frenzylk’ was ‘frenzy …’, and the correct typed response was ‘lk’. Responses were considered correct if the letters were in the right position. The learning phase was completed once, whilst cued recall was completed twice: once immediately following learning, and repeated again following a delay containing the same cues.

#### Anterograde task

**Figure 1:**
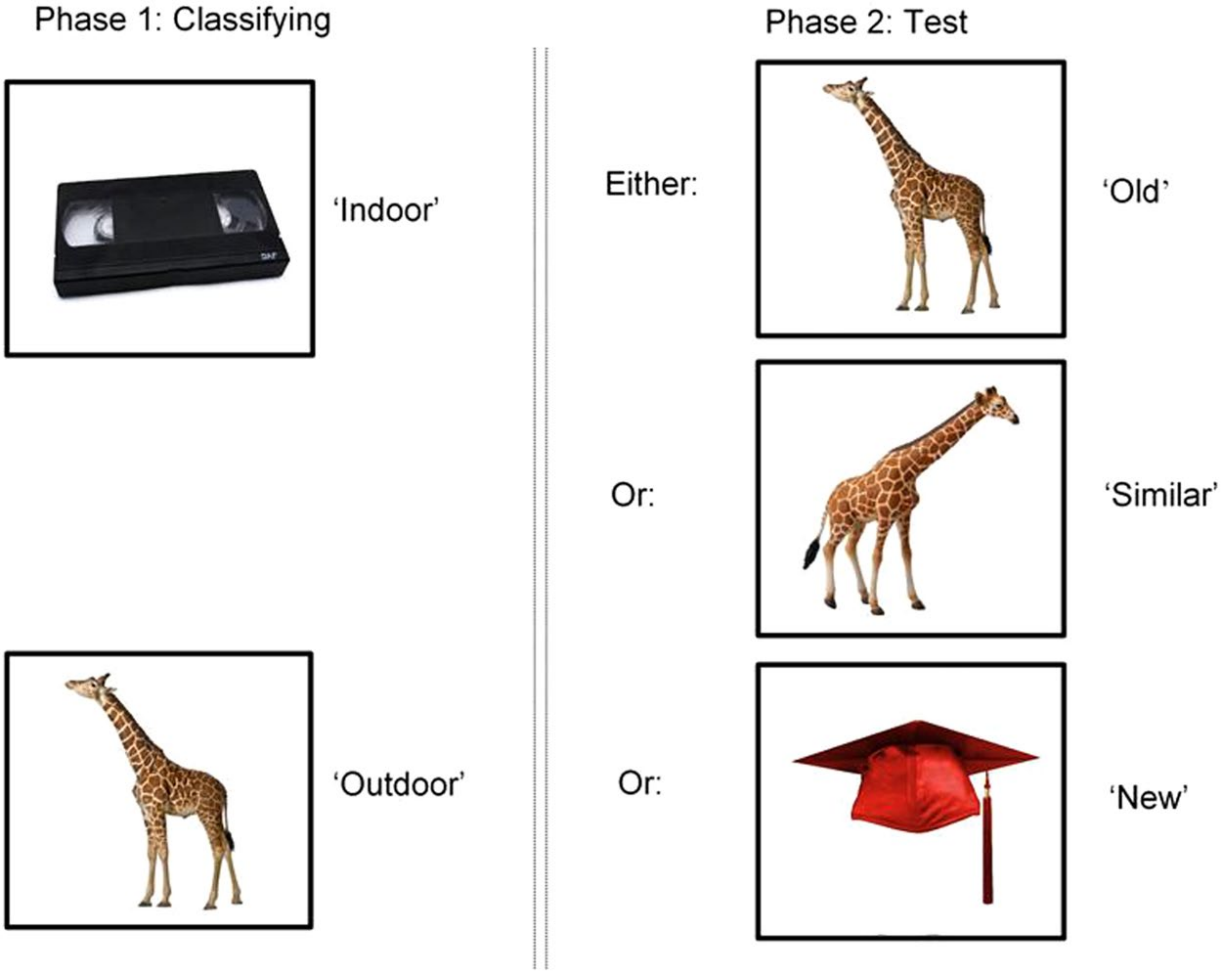
Phase one and two of the MST. Phase one involved classifying objects as either ‘indoor’ or ‘outdoor’. Phase two followed immediately after, and assessed participant’s memory for the objects they had seen in phase one by presenting either the object again (target), a perceptually similar but not identical object (lure), or a completely new object (foil). Only one of these three categories were presented; for example, the target was not presented again if the similar object had already been presented. Figure produced with author permission from Stark *et al*.^14^. Images accessed at http://faculty.sites.uci.edu/starklab/mnemonic-similarity-task-mst/.

To measure acute memory performance, a Mnemonic Similarity Task (MST)^14^ measured the acute impairment caused by alcohol immediately after intoxication using two phases. During phase one, participants observed 128 images of objects on the screen, and were required to press a key classifying them as ‘indoor’ or ‘outdoor’. Phase two followed, and tested memory recognition as participants were required to identify 192 objects on the screen as either ‘old’, ‘similar’, or ‘new’. Of these 192 objects: 64 were identical repetitions of objects from phase one (targets); 64 were objects that were perceptually similar, yet not identical to those observed in phase one (lures); and 64 were completely new objects not previously observed (foils). Following a randomisation process, participants completed either one of two stimulus sets on session one, and completed the alternate set on session two. This paradigm is illustrated below, in Fig. 1. As suggested^14^, traditional recognition memory was calculated by subtracting the percent of foils falsely identified as ‘old’ from the percent of targets accurately identified as ‘old’. Furthermore, behavioural pattern separation (BPS score) was also calculated by subtracting percent of foils falsely identified as ‘similar’ from the percent of lures correctly identified as ‘similar’, which gives an index of the ability to distinguish between two similar patterns. The MST was completed twice between two sessions, and contained independent, non-repeated stimuli in both.

#### Alcohol content measures

Breathalyser readings were taken using digital alcohol detector devices (Kx6000 s, AlcoSafe) throughout session one and two. This provided an estimate of breath alcohol content (BrAC) through vapour in breath.

#### Questionnaires

The Alcohol Use Disorders Identification Test (AUDIT^17^) and the Rapid Alcohol Problems Screen (RAPS4^18^) were used to measure levels of harmful drinking and mild dependence.

Participants were asked a single question to identify whether they typically experienced blackouts after drinking alcohol: “Have you had blackouts (“loss of memory” without passing out) as a result of drinking?”, and were required to answer “No, never; Sometimes; Often; or Almost every time I drink”.

### Procedure

**Figure 2:**
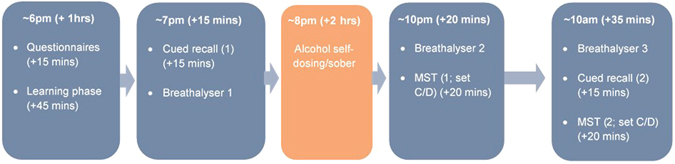
Schematic illustration of the sequence of memory tasks participants underwent during each session, accompanied by approximate timings.

Participants attended two sessions on two consecutive days that were approximately 16 hours apart. Session one took place at 6 pm, and lasted approximately four hours. Session two took place at 10 am the following morning, and lasted approximately 45 minutes. All procedures and approximate timings are schematically illustrated by Fig. 2.

#### Session one

At the beginning of session one, participants were screened and provided their informed consent. They then provided demographic information and completed all questionnaire measures. Participants then completed the learning phase of the retrograde task, immediately followed by cued recall. Upon finishing this task, at around 8 pm, participants self-dosed with alcohol ad libitum for two hours. During this time the sober condition engaged in drinking non-alcoholic beverages of their choice. Following the two hours of drinking, participants completed the memory anterograde task. Two breathalyser recordings were taken: once before drinking, and once following drinking.

#### Alcohol Administration

During the study session, alcohol was self-dosed and administered over a two hour period and in a social context. During this period, the dose of alcohol was continuously estimated by participants. Participants were permitted to consume further alcohol upon finishing testing after the experimenter had left, but were requested to count any further units consumed. Any additional units consumed by participants after session one were recorded at the start of session two the next morning. The sober condition was required to abstain from consuming alcohol between sessions.

#### Session two

The next morning the test session started at +18 hours following encoding, a breathalyser reading was taken and a self-reported estimation of alcohol consumption between sessions was provided. This was followed by the second, repeated phase of cued recall, and completion of the alternative stimulus set for the anterograde task. Upon completing all procedures, participants were debriefed and financially compensated for their time.

### Statistical Analysis

Data were analysed using the Statistical Package for Social Sciences (SPSS) version 23. Assumptions of normality were checked, as was homogeneity of variance using Levene’s test.

Group performance on both the consolidation and anterograde tasks were analysed using 2 × 2 mixed ANOVAs with group (alcohol, sober) as the between-subjects factor and session (session one, session two) as the within-subjects factor. For the anterograde task, traditional recognition memory was calculated by subtracting the percent of foils identified as ‘old’ from the percent of targets identified as ‘old’, and BPS score was calculated by subtracting percent of foils identified as ‘similar’ from the percent of lures identified as ‘similar’.

Non-parametric data were analysed using the Mann-Whitney *U* test, or Chi-square test where data are categorical. Post-hoc analyses were adjusted using the Bonferroni correction to correct for multiple comparisons. Associations were assessed with Pearson’s correlations, unless assumptions of normality were violated, where a Spearman’s correlation was used.

## Results

### Demographics and Alcohol Use

The sober and alcohol conditions did not significantly differ in age, BMI, alcohol use, or incidence of blackouts. Likewise, conditions did not differ in diagnosis of either mental illness or alcoholism in a first degree relative. Conditions did significantly differ in years of education with greater years of education in the alcohol group, and use of oral contraceptives among female participants which was significantly greater in the alcohol group (see Table 1).

**Table 1:** Participant Demographics (Means and SD) Between Conditions.

	Condition			
	Sober (n = 45)	Alcohol (n = 43)	t/χ^2^	*p*
Age	22.53 (7.28)	24.02 (9.29)	−0.84	0.404
Education (years)	14.91 (2.64)	16.15 (1.71)	−2.58	0.011*
Body mass index (BMI)	23.15 (4.66)	24.11 (4.64)	−0.95	0.344
Alcohol used (years)	5.99 (5.15)	7.93 (8.05)	−1.29	0.202
Alcohol use (days in month)	5.25 (3.61)	6.97 (4.62)	−1.83	0.071
Amount used in typical session (g)	73.99 (48.95)	63.91 (36.54)	1.09	0.278
Days since last use	15.67 (54.05)	4.40 (5.21)	1.45	0.151
Amount last used (g)	58.84 (57.58)	60.36 (52.83)	−0.13	0.899
AUDIT score	10.73 (5.99)	10.26 (4.95)	0.41	0.685
RAPS4 score	6.02 (1.08)	5.91 (0.90)	0.55	0.587
Blackout Questionnaire score	3.51 (1.47)	3.65 (1.34)	−0.47	0.643
Oral Contraceptive, (*n*)	6	14	5.37	0.020*
Tobacco use, (*n*)	22	21	0.00	0.996

The self-report measures: high AUDIT scores indicate increased harmful drinking behaviour; high RAPS4 scores indicate higher alcohol dependence; high Blackout Questionnaire scores indicate increased experience of blackouts when intoxicated.

### Intoxication: Dose and Breathalyser Readings

During the alcohol administration period during session one, participants in the alcohol condition drank an average of 53.38 g (*SD* = 31.09) of alcohol. The total amount of alcohol consumed, including both the estimates during session one and participants self-reported additional units consumed after session one, averaged at 82.59 g (*SD* = 50.37) of alcohol.

**Figure 3:**
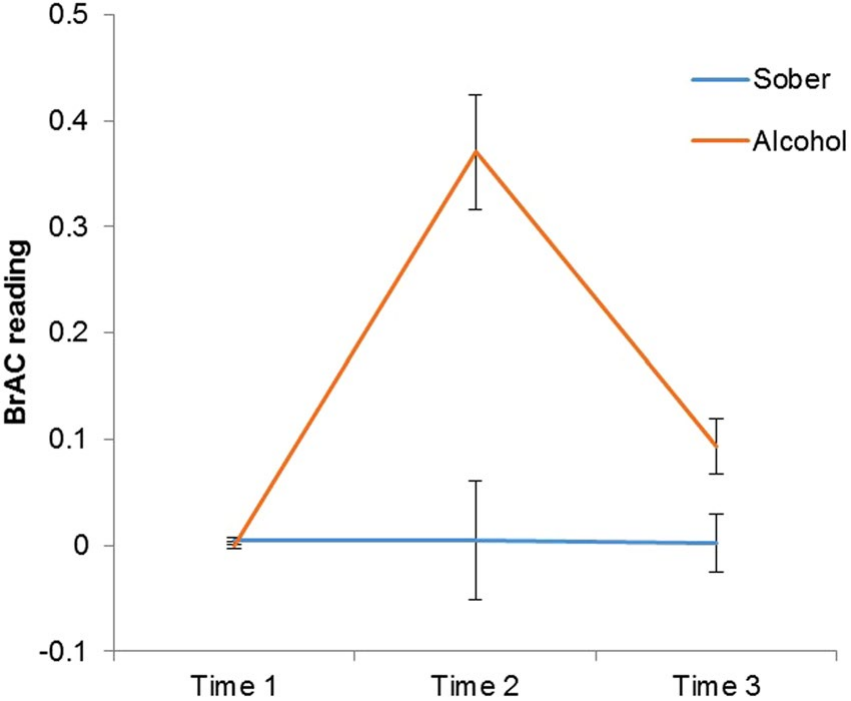
The average breath alcohol content (BrAC) readings for both the alcohol and sober conditions at three different time points (before drinking; following drinking; the morning after drinking) throughout the duration of the experiment, with standard error bars. There were significant differences between groups following drinking, both at time two (*p* < 0.001) and time three (*p* < 0.001). There were no significant group differences before drinking.

The breath alcohol content (BrAC) recordings for both conditions at each time point are shown in Fig. 3. Due to the non-parametric nature of this data, a Mann-Whitney U test was conducted to confirm the difference in readings between conditions at each stage of intoxication. No group differences were observed at baseline before intoxication (breathalyser one) (*U* = 946, Z = −0.98, *p* = 0.328). BrAC recordings were significantly higher in the alcohol condition (*Mdn* = 0.13, *IQR* = 0.34) than the sober condition (*Mdn* = 0.00, *IQR* = 0.00) immediately following intoxication (breathalyser two) (*U* = 27, Z = −8.40, *p < *0.001). BrAC recordings between conditions were also significantly different the morning following intoxication (breathalyser three) (*U* = 624.5, Z = −4.16, *p < *0.001), as the alcohol condition exhibited higher readings (*Mdn* = 0.00, *IQR* = 0.20) than the sober condition (*Mdn* = 0.00, *IQR* = 0.00).

### Retrograde task

**Figure 4:**
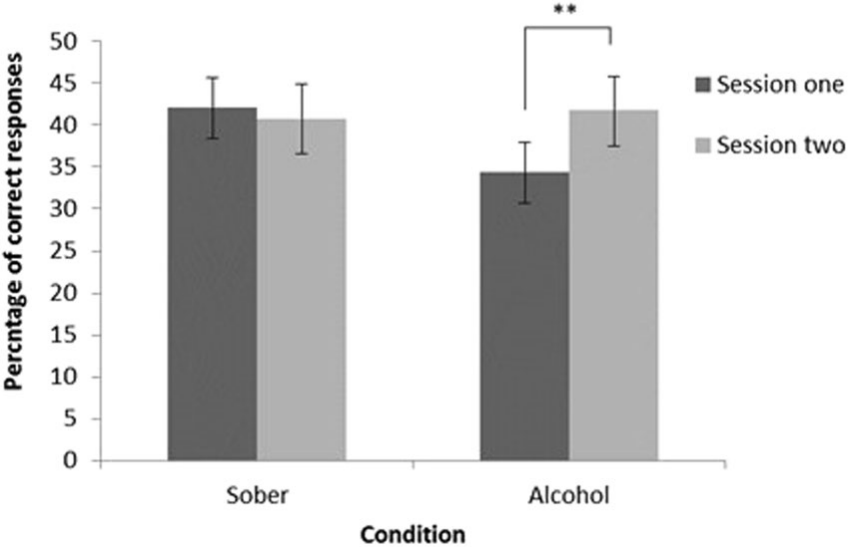
The proportion of correct responses during the cued recall task between the alcohol and sober conditions during session one and two. A significant increase in correct responses between sessions was found in the alcohol condition only (*p* = 0.008). There were no significant group differences.

When assessing cued recall, a 2 × 2 mixed ANOVA revealed a significant session × group interaction (F (1, 82) = 237.29, *p* < 0.001). Bonferroni corrected post-hoc comparisons revealed no significant differences between sessions in the sober condition (F (1, 82) = 0.22, *p* = 0.641), but a significant difference between sessions in the alcohol condition (F (1, 82) = 7.27, *p* = 0.008), where individuals made more correct responses during session two than session one (Fig. 4). There were no significant main effects of session (F (1, 82) = 2.57, *p* = 0.113) or group (F (1, 82) = 0.43, *p* = 0.516) on performance.

### Anterograde task

**Figure 5:**
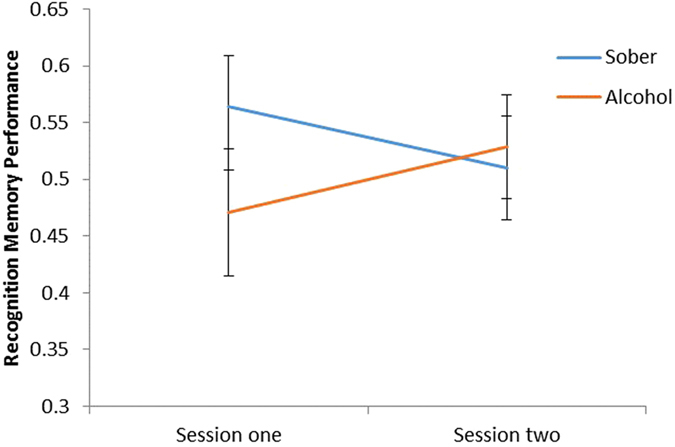
The interaction between session and condition on recognition memory performance during the acute memory task. A significant decline in performance was found between sessions in the sober group only (*p* = 0.021). There were no significant group differences.

During the anterograde MST task, the proportion (%) of each response type to each stimuli type during phase two between the groups can be seen in Table 2. A 2 × 2 mixed ANOVA for recognition memory performance (percentage of targets identified as ‘old’ minus foils identified as ‘old’) during the MST revealed a significant session × group interaction (F (1, 85) = 4.62, *p* = 0.034) (Fig. 5). Bonferroni corrected post-hoc comparisons revealed that there was a significant difference in the sober condition between sessions (t (44) = 2.40, *p* = 0.021), where performance declined from session one (*M* = 0.57, *SD* = 0.30) to session two (*M* = 0.52, *SD* = 0.29); the alcohol condition did not differ between sessions (t (42) = −1.23, *p* = 0.227). There was no significant main effect of group (F (1, 85) = 0.39, *p* = 0.532) or session (F (1, 85) = 0.01, *p* = 0.935) on performance.

**Table 2:** Hit Rates for Each Stimulus and Response Type, as well as BPS Scores (Means and SD) During the Memory Anterograde task in Session One and Two between the Alcohol and Sober Condition.

		Target			Lure			Foil			BPS score
		Old	Similar	New	Old	Similar	New	Old	Similar	New	
Alcohol (n = 43)	Session one	0.61 (0.26)	0.18 (0.14)	0.22 (0.25)*	0.41 (0.18)	0.32 (0.16)*	0.27 (0.24)*	0.13 (0.13)	0.20 (0.13)	0.67 (0.22)	0.12 (0.15)
	Session two	0.65 (0.27)	0.20 (0.20)	0.16 (0.21)*	0.41 (0.19)	0.39 (0.19)*	0.20 (0.20)*	0.11 (0.14)	0.23 (0.20)	0.66 (0.26)	0.17 (0.20)
Sober (n = 45)	Session one	0.70 (0.23)*	0.19 (0.20)	0.12 (0.15)*	0.41 (0.16)	0.40 (0.22)	0.18 (0.16)*	0.11 (0.13)	0.23 (0.19)	0.65 (0.23)	0.16 (0.20)
	Session two	0.65 (0.21)*	0.17 (0.13)	0.18 (0.18)*	0.39 (0.14)	0.36 (0.20)	0.25 (0.18)*	0.11 (0.11)	0.19 (0.15)	0.71 (0.17)	0.16 (0.19)

*Significant at *p* < 0.05.

**Significant at p < 0.001.

Accurate hit rates are reflected by correctly responding to the stimulus targets, lures, or foils as “old”, “similar”, or “new”, respectively, with higher scores reflecting proportion of hits for each. False alarms to both lures (“old”|lure) and foils (“old”|foil), can also be observed, as well as incorrect “similar” responses to targets (“similar”|target) and foils (“similar”|foil).

A 2 × 2 mixed ANOVA on BPS scores (see Table 2) (percentage of lures identified as ‘similar’ minus foils identified as ‘similar’) did not show a session × group interaction (F (1, 85) = 2.01, *p* = 0.150), nor were there any main effects of condition (F (1, 85) = 2.54, *p* = 0.115) or session (F (1, 85) = 0.10, *p* = 0.753).

### Correlations

**Figure 6:**
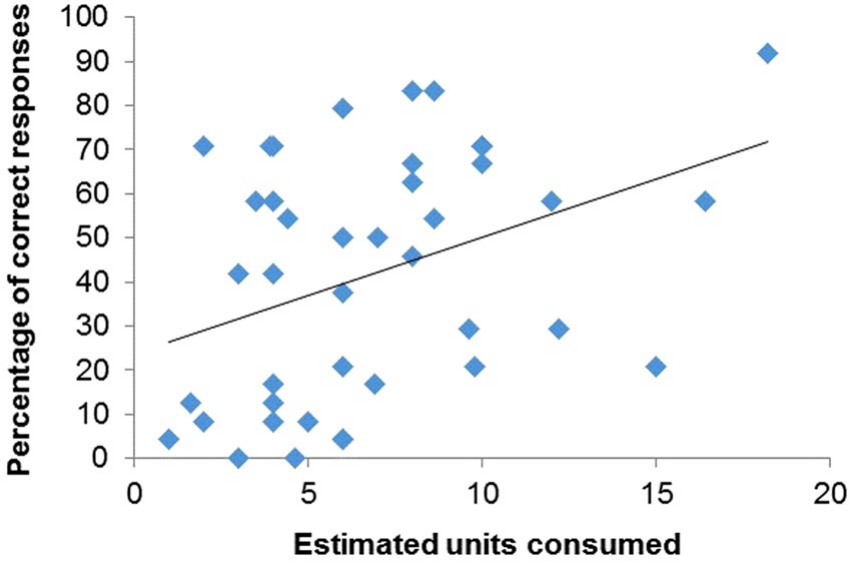
A significant positive correlation between the estimated units consumed in session one and proportion of correct responses during cued recall in session two within the alcohol condition only (*p* = 0.015). The amount of alcohol consumed was positively related to scores on the retrograde memory test the following day, as individuals who consumed more shown enhanced performance.

A Pearson’s correlation revealed a significant positive relationship between the units of alcohol consumed during session one (during experimentation) and next day performance on the retrograde task: those in the alcohol condition who consumed more units of alcohol made more correct responses the following day (r = 0.38, n = 41, *p* = 0.015) (Fig. 6).

To investigate whether retrograde facilitation was directly related to anterograde impairment in the alcohol condition, Pearson’s correlations were conducted between performance on the anterograde task in session one (completed when intoxicated) with next day performance on the cued recall. No significant relationship was observed between recognition memory (r = 0.14, n = 41, *p = *0.373) or BPS scores (r = −0.07, n = 41, *p* = 0.69) in session one with subsequent cued recall performance the following day.

## Discussion

The current study set out to examine whether retrograde facilitation of memory following alcohol is observed in a naturalistic setting. As hypothesised, people in the alcohol condition exhibited improved memory on the retrograde task the following day after consumption compared to before they had drunk, but no improvement was observed in the sober group. The improvement in the alcohol group was related to the dose of alcohol consumed. The study also found poorer recognition memory performance on the anterograde task on the second day of testing in the sober group compared to the first, but no difference in the alcohol group across the two sessions.

The results of this study support the notion that alcohol can facilitate memory for previously learned information. By replicating retrograde facilitation in people drinking in their own homes, this finding extends past laboratory findings^2–5^ to suggest that these controlled studies have ecological validity. In addition to this, a correlation suggested that within the alcohol condition, the total amount of alcohol consumed during session one was positively related to performance on the retrograde task the following day. Thus, individuals in the alcohol condition who consumed more alcohol made more correct responses during cued recall the subsequent morning. This is similar to previous findings in the laboratory administering three doses of alcohol^2^, where the two highest doses of alcohol caused significantly greater enhancement of memory for information learnt prior to drinking compared with the lowest dose. The current study thus suggests that alcohol dose can have a gradient effect on consolidation, implying that as greater quantities of alcohol are consumed, there is less retroactive interference. This may be explained by the creation of a neural state which better facilitates cellular and systems consolidation as dose increases. It is also interesting to note that performance on the retrograde task was better on the day following alcohol use in the alcohol group. Recent work has suggested that sleep may facilitate access to memory traces that are initially too weak to recover on a recall task such as this^19^, and these data may suggest that alcohol might be interacting with this process, rather than simply reducing forgetting.

The idea that alcohol prevents subsequent encoding and has an impact on memory performance the following day would be further supported by observing a relationship between extent of anterograde amnesia in session one and subsequent memory performance, yet this was not found. In the absence of this additional correlation, it is difficult to definitively conclude that reduced retrograde interference is solely responsible for the memory improvements found. Our anterograde task that measured alcohol intoxication was not sensitive enough to the memory impairing effects of alcohol, as performance in the alcohol condition did not change between sessions on this measure, despite the amnestic effects of alcohol. Nonetheless, alcohol dose was significantly related to improved memory performance the subsequent day, suggesting the gradient effect of alcohol intoxication on consolidation.

In light of the previous point, future work should make attempts to empirically rule-out alternative neurobiological explanations for retrograde facilitation by controlling for sleep. A large proportion of the literature has ignored the influence of sleep on consolidation, as many studies that report retrograde facilitation have comprised a period of sleep between sessions^2, 4, 5^. Since alcohol can increase the proportion of slow wave sleep (SWS) the night following drinking^20^, and SWS is crucial for declarative memory consolidation^15^, it is possible that retrograde facilitation may partially be a consequence of increased SWS, as opposed to a reduction in interference alone. It is important to rule out this explanation by controlling for SWS between sessions using polysomnography, and taking measures of sleep quality and sleep architecture.

The ability to reproduce this subtle effect on memory consolidation in an environment with less experimental control is not only important for enhancing the ecological validity of laboratory findings, it is also informative on how alcohol interacts with memory processes in the environments where alcohol consumption generally occurs. In essence, this enables researchers to make more generalisable claims about the cognitive consequences of alcohol consumption in environments where alcohol is frequently consumed, which is important for advising public health interventions. Rather than the wholly negative effect that alcohol is presumed to have on memory, this study suggests that there may be some subtle enhancing effects, that may be informative in future for developing novel pharmacotherapies and tailored cognitive enhancing interventions. Clearly, from a public health perspective, the communication of these subtle and highly constrained positive effects of alcohol on memory should be carefully considered in the context of the wide-ranging longer term cognitive impairments associated with frequent heavy consumption of this drug.

Performance on the memory anterograde task revealed a decline in performance of the sober group from session one to session two. There was not a significant increase in performance in the alcohol group across the two sessions, which might have been expected considering the acute memory impairing effects of alcohol. It may be that the task was not sensitive enough to pick up the alcohol-induced impairment on session one, although there was a tendency towards this difference. Alcohol has been suggested to have relatively confined effects on memory at the dose participants were tested at in the current study, which affects memory for peripheral rather than central material^21, 22^, and with memory for pictures less impaired by alcohol than memory for words.

The poorer performance on the anterograde task in session two in the sober group might be explained by considering the impact of interference on memory. It is possible that during the second session the sober condition experienced proactive interference from completing the task the prior evening, meaning that the pictures they had encoded then were making the task more difficult the following day. The alcohol group would not be subject to this interference. Although the current study used two stimulus sets between sessions to reduce practice effects, the within subjects design could have elicited proactive interference so that the temporally close sessions could have caused interference within the sober condition. This possible explanation would also align with the assumptions made by theories explaining retrograde facilitation: memory for previously learned information is enhanced via a reduction in encoding new information following alcohol consumption, which could explain why the alcohol condition did not experience proactive interference from the memory anterograde task.

In addition to the above, analysis of the behavioural pattern separation score taken from the memory anterograde task did not reveal any significant differences in performance between conditions or sessions. This score is believed to give a more sensitive measure of participants’ pattern separation performance, since it corrects for any response biases that may be present in the traditional measure of recognition memory^14^. Including an additional phase of the memory anterograde task *before* intoxication would have provided a baseline to compare performance with, both immediately after intoxication and the following morning, but was not possible in this study due to time constraints.

There are inevitable limitations of a naturalistic design. We allowed participants the freedom to consume the alcoholic beverage and quantity of their choice, but then were largely reliant on the participants’ estimates for amount of alcohol consumed, which can be inaccurate particularly as alcohol is a memory impairing drug^23^. One method to improve this would be to include objective and remote measurements of blood alcohol concentrations (BAC) throughout the duration of the study, such as fitting participants with a continuous transdermal alcohol monitor that continually assesses BAC through perspiration. The inclusion of this objective measure would enable researchers to assess the trajectory of BAC over a session of drinking and compare this with cognitive performance, and allow for comparisons between participants; in relation to dose, peak intoxication, and length of time until BAC readings decline back to zero. Such a measure would enable researchers to control for these differences in intoxication between participants in studies with naturalistic designs, which could be included in future research.

Related to the point above, participants were tested in their home environment, which arguably is not the most prototypical environment in which drinking takes place. Efforts to test in more naturalistic settings, such as a bar or club, were met with heavily resistance from these businesses, and hence the most available option was to test participants in their homes during a drinking session that preceded a night out.

Overall, the current research has provided compelling evidence that retrograde facilitation can occur outside a laboratory setting, in a natural environment in which alcohol is frequently consumed. Furthermore, correlative evidence suggests that amount of alcohol is related to rate of consolidation; where larger doses improve memory more than smaller doses, putatively due to reduced interference at higher doses. Initially, these findings would indicate that alcohol could cause disruptions to hippocampal encoding, as the current study suggests alcohol interferes with the ability to store information encountered following intoxication. However, without the confirmatory evidence from the anterograde task, this claim should be made with caution. Nonetheless, this finding in naturalistic settings has greater generalisability than laboratory studies to real-life situations where alcohol is frequently consumed; and potential clinical implications, for example reducing peritraumatic alcohol use.
